# Prediction of Antimalarial Drug-Decorated Nanoparticle Delivery Systems with Random Forest Models

**DOI:** 10.3390/biology9080198

**Published:** 2020-07-30

**Authors:** Diana V. Urista, Diego B. Carrué, Iago Otero, Sonia Arrasate, Viviana F. Quevedo-Tumailli, Marcos Gestal, Humbert González-Díaz, Cristian R. Munteanu

**Affiliations:** 1 Department of Organic Chemistry II, University of Basque Country (UPV/EHU), Sarriena w/n, 48940 Leioa, Spain; diana_urista_marquez@hotmail.com (D.V.U.); sonia.arrasate@ehu.es (S.A.); humberto.gonzalezdiaz@ehu.es (H.G.-D.); 2 RNASA-IMEDIR, Computer Science Faculty, CITIC, University of A Coruna, Campus Elviña s/n, 15071 A Coruña, Spain; d.bcarrue@gmail.com (D.B.C.); i.otero.coto@gmail.com (I.O.); viviana.quevedo@udc.es (V.F.Q.-T.); marcos.gestal@udc.es (M.G.); 3 Universidad Estatal Amazónica UEA, Km. 2 1/2 vía Puyo a Tena (paso lateral), Puyo 160150, Pastaza, Ecuador; 4 Biomedical Research Institute of A Coruña (INIBIC), Hospital Teresa Herrera, Xubias de Arriba 84, 15006 A Coruña, Spain; 5 IKERBASQUE, Basque Foundation for Science, Alameda Urquijo 36, 48011 Bilbao, Spain; 6 Basque Centre for Biophysics CSIC-UPVEHU, University of Basque Country UPV/EHU, Barrio Sarriena, 48940 Leioa, Spain

**Keywords:** decorated nanoparticles, drug delivery, antimalarial compounds, big data, Perturbation Theory, Machine Learning, ChEMBL database

## Abstract

Drug-decorated nanoparticles (DDNPs) have important medical applications. The current work combined Perturbation Theory with Machine Learning and Information Fusion (PTMLIF). Thus, PTMLIF models were proposed to predict the probability of nanoparticle–compound/drug complexes having antimalarial activity (against Plasmodium). The aim is to save experimental resources and time by using a virtual screening for DDNPs. The raw data was obtained by the fusion of experimental data for nanoparticles with compound chemical assays from the ChEMBL database. The inputs for the eight Machine Learning classifiers were transformed features of drugs/compounds and nanoparticles as perturbations of molecular descriptors in specific experimental conditions (experiment-centered features). The resulting dataset contains 107 input features and 249,992 examples. The best classification model was provided by Random Forest, with 27 selected features of drugs/compounds and nanoparticles in all experimental conditions considered. The high performance of the model was demonstrated by the mean Area Under the Receiver Operating Characteristics (AUC) in a test subset with a value of 0.9921 ± 0.000244 (10-fold cross-validation). The results demonstrated the power of information fusion of the experimental-centered features of drugs/compounds and nanoparticles for the prediction of nanoparticle–compound antimalarial activity. The scripts and dataset for this project are available in the open GitHub repository.

## 1. Introduction

Drug-decorated nanoparticles (DDNPs) are among the most interesting nanomaterials, with a broad range of medical applications. Many of them are used in drug delivery systems for different types of chemical compounds. These systems have numerous advantages, since there are countless combinations of drugs and nanoparticles that can be effective in treating different conditions. At the same time, they have some weaknesses. For example, the synthesis of nanoparticles can sometimes be expensive, or it can involve a lot of time that can increase with the number of samples. For this reason, in order to improve the possibility of forming effective pairs, there is a need for computational models.

Recently, some researches have been focusing on finding DDNPs that show antimalarial properties. For instance, silver and gold nanoparticles, that were synthesized from leaf and bark extracts of Myrtaceae, exhibited an effective antiplasmodial activity [1], and exopolysaccharide coated ZnO nanoparticles (EPS-ZnO NPs) presented functional effects against malaria vectors [2]. Therefore, this study aims to design a useful computational model that allows a good prediction of the antimalarial activity of varied drug–nanoparticle pairs.

Moreover, a brand new method for data fusion in nanotechnology, bio-molecular sciences, chemistry and big data analysis has been proposed in different works: it integrates Perturbation Theory (PT) and Machine Learning (ML) [3,4,5,6,7,8,9,10,11,12,13], using distinct PT operators to analyze changes in the varied non-structural and structural conditions of a test at once (PTML). A few of these PT operators represent the generalization of a classic cheminformatics approach introduced by Corwin Hansch [14]. He noticed the significant potential of using predictive methodologies to resolve multivariate questions in medicinal chemistry. Hansch’s classic approach allows one to search for models with multiple physicochemical conditions so as to foretell the biological activity of compounds, and these models possibly include quadratic and/or linear terms. In this process, which is a Linear Free Energy Relationship (LFER) model, most of the terms are physicochemical parameters linked with the free energy of drug ionization, binding, transport, etc. In addition, because we are fusing the information (IF) of drugs and nanoparticles, the model becomes a PTMLIF (PTML + IF).

As an illustration, the logarithmic term (logP) of the octanol/water partition coefficient (P) is presented as an estimate of the free energy of drug transport and molecular lipophilicity [15]. We can approximate logP values via chemical fragment methods (such as CLogP), or via atomic methods (such as ALogP or XLogP) [16,17]. The logarithmic terms of acidity constants (pKa) are connected to the free energy of drug ionization. Additionally, to account for more molecular properties, we can use different parameters like Polar Surface Area (PSA). Generally, for a given molecule *m_i_*, we can utilize as input for the model several types of molecular properties, taking into account measures of molecular polarizability, lipophilicity, electronegativity, etc. [17,18]. We can define these models as:

(1) $$f\left(\varepsilon_{i}\right)=\overset{kmax}{\underset{k=1}{\sum }}a_{k}\cdot D_{k}\left(m_{i}\right)+\overset{kmax}{\underset{k=1}{\sum }}b_{k}\cdot D_{k}{\left(m_{i}\right)}^{2}+e_{0}$$

where *ε_i_* is the biological activity of the molecule *m_i_*, *f*(*ε_i_*) is a function of the variable *ε_i_*, *D_k_*(*m_i_*) are the molecular descriptors of *m_i_*, and *a_k_* and *b_k_* are the coefficients. This classical model works to account for changes in the chemical structure of the drug/compound using the molecular descriptors, but it does not take into account the result regarding the drug activity of perturbations in multiple experimental conditions (c_j_). These include assay conditions or changes in drug chemical structure, such as c_0_ = the biological parameter used (CC_50_: the ratio of the 50% cytotoxic concentration, IC_50_: inhibition concentration, etc.), c_1_ = organism, c_2_ = cell name, c_3_ = assay organism, etc. An example is the large datasets found in the public database ChEMBL [19,20,21,22,23,24,25]. We used PTML methods to analyze a large set of over 50,000 preclinical assays of drugs. These assays incorporate drugs targeting Plasmodium. The PTML model proceeds from the classic LFER approach for drug activity. We combined the use of eight Machine Learning methods with feature selection in order to obtain a more accurate classifier for our task. 

## 2. Materials and Methods

**Figure 1:**
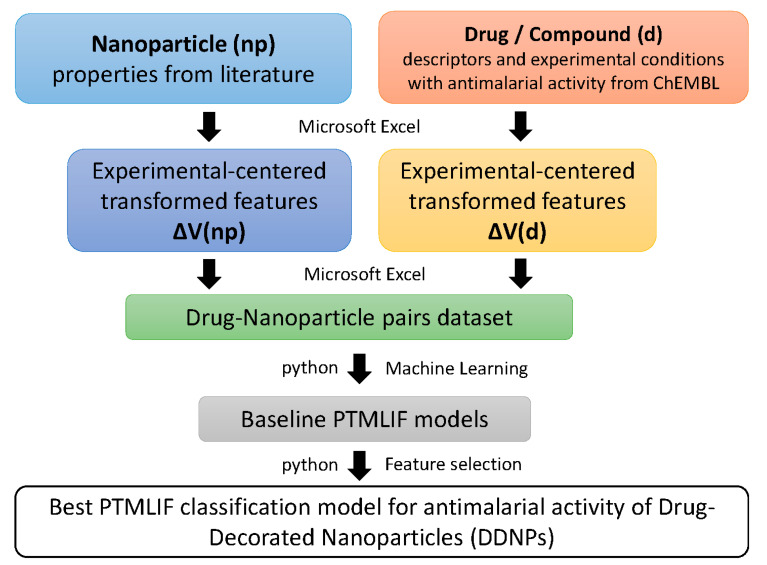
Workflow for development of the Perturbation Theory-Machine Learning (PTML) model.

Figure 1 presents the methodology used to build the PTMLIF classifier for the antimalarial activity of DDNPs. The methodology flow contains the following steps: (1) Get 23 properties of nanoparticle and anti-malaria drugs/compounds from the literature and public databases as initial molecular descriptors; (2) Fuse information about experimental conditions and the properties of anti-malaria drugs/compounds and nanoparticles, using an experimental-centered transformation of the original features (Box–Jenkins Moving Average operators); (3) Integrate drug/compound and nanoparticle data into the study dataset; (4) Build the baseline PTMLIF models using default parameters of the ML methods; (5) Improve the performance of the best classifier by using only the most important features (feature selection).

The initial molecular descriptors were Mw, PSA and ALOGP (3 descriptors for the ChEMBL compounds/small molecules), and NMUnp, Lnp, Vnpu, Enpu, Pnpu, Uccoat, Uicoat, Hycoat, AMRcoat, TPSA(NO)coat, TPSA(Tot)coat, ALOGPcoat, ALOGP2coat, SAtotcoat, SAacccoat, SAdoncoat, Vxcoat, VvdwMGcoat, VvdwZAZcoat and PDIcoat (20 descriptors for nanoparticles). The following abbreviations were used: Mw = molecular weight; PSA = polar surface area; ALOGP = logarithmic term of the octanol/water partition coefficient; np = nanoparticle; npu = nanoparticle elemental unit (Al, SiO2, etc.); NMU = number of monomeric units in the np; V = average of atomic Van der Waal Volume for all atoms in the npu (<V(cm^3^/mol)>); E = electronegativity; P(A3) = atomic polarizability; L = np large (experimental data); UC = uncoated nanoparticles; NMU = number of monomer units; HMT = Hexamethylenetetramine; TMAOH = Tetramethylammonium Hydroxide; DMEM = Dulbecco’s modified Eagle’s medium; coat = np coating; Uc = unsaturation count; Ui = unsaturation index; Hy = hydrophilic factor; AMR = Ghose–Crippen molar refractivity; TPSA(NO) = topological polar surface area using N,O polar contributions; TPSA(Tot) = topological polar surface area using N,O,S,P polar contributions; ALOGP2 = squared Ghose–Crippen octanol/water partition coefficient (logP^2); SAtot = total surface area from P_VSA-like descriptors; SAacc = surface area of acceptor atoms from P_VSA-like descriptors; SAdon = surface area of donor atoms from P_VSA-like descriptors; Vx = McGowan volume; VvdwMG = van der Waals volume from McGowan volume; VvdwZAZ = van der Waals volume from Zhao–Abraham–Zissimos equation; PDI = packing density index.

### 2.1. ChEMBL Data Pre-Processing

We got the results of several preclinical assays from ChEMBL. The experimental measure, ε_ij_(d), used to quantify the biological activity of the i-th molecule (m_i_) over the j-th objective, represents the outcome of every assay. The values of ε_ij_(d) rely on the structure of the compound, and also on certain limit conditions that mark off the properties of the assay **c**_j_(d) = (c_0_(d), c_1_(d), c_2_(d), …, c_n_(d)). The first c_j_(d) is c_0_(d) = the biological activity; we used drugs with CC_50_, EC_50_ and IC_50_. Other conditions are c_1_(d) = organism, c_2_(d) = cell name, c_3_ = assay organism, c_4_(d) = assay strain, etc. (see Table 1). We used classification techniques because the values ε_ij_(d) are not exact numbers in some cases. Furthermore, we discretized the values in this way: for IC_50_ and EC_50_ f(v_ij_(d))obs = 1 when v_ij_ < cutoff and desirability of the biological activity parameter D(c_0_(d)) = −1 (see Table 2); for CC_50_, f(v_ij_(d))obs = 1 when v_ij_ > cutoff and desirability D(c_0_(d)) = 1, if not f(v_ij_(d))obs = 0. The desirability D(c_0_(d)) = 1 or −1 denotes that the parameter measured decreases or increases directly with a biological effect, which can be desired or not. Finally, we calculated the deviations of each condition for all drugs/compounds.

### 2.2. Nanoparticle Data Pre-Processing

From the literature, we collected the outcomes of many nanoparticles, and the measure ε_ij_ expresses the result of each of them. The values of ε_ij_(np) depend on different properties of the nanoparticle, and also on some boundary conditions that delimit the characteristics of the assay c_j_ (np) = (c_0_(np), c_1_(np), c_2_(np), …, c_n_(np)) (see Table 3). Again, the first c_j_(d) = the biological activity, and we only used nanoparticles with CC_50_, EC_50_ and IC_50_, so that they could match with the biological activities of the drugs/compounds. Other conditions are c_1_(np) = cell name, c_2_(np) = nanoparticle shape, c_3_(np) = nanoparticle medium and c_5_ = surface coating. Additionally, we discretized the values in the same way that we did with drugs/compounds (see Table 4). In the end, we determined the deviations of every c_j_ for all nanoparticles. 

### 2.3. Combine Data PRE-Processing

Once both databases were done, we combined them by doing pairs with the same experimental conditions, for example, a CC50 with a CC50 nanoparticle. In addition, we used the same method to discretize each formed pair. Thus, a dataset of 107 input features and 249,992 examples will be used to build ML classification models. The positive (1) and negative control cases (0) were assigned as follows: if desirability function d(c0) = −1, then c_ij_ = 1 when ε_ij_ < 100 nM or ε_ij_ < average <ε_ij_> for properties not measured in nM. In addition, if d(c_0_) = 1, 0, then c_ij_ = 1 when ε_ij_ > average value <ε_ij_>. An extra input feature (prob = probability) was created as the probability of c0 for compound–nanoparticle pairs (count of the number of compound–nanoparticle pairs for each c0 activity type/total number of pairs). The name of the final features in the dataset has the format [d_/np_][original descriptor name]([experimental condition]). For example:

- d_DPSA(c2) = difference (D) between original values of PSA descriptor and the mean of PSA values in experimental condition c2 (for drugs/compounds, d_);
- np_DLnp(c4) = difference between Lnp value and the mean of Lnp values in experimental condition c4 (for nanoparticles, np_).

### 2.4. Machine Learning Methods

The study is done using eight Machine Learning scikit-learn classifiers to find the best classifier able to predict the probability of a nanoparticle–compound pair highly express antimalarial activity:

1. KNeighborsClassifier = KNN—k-nearest neighbors: one of the most well-known non-parametric classifiers in the ML field. It assigns an unclassified sample to the same class as the nearest of the k samples in the training set [26];
2. SVC(linear) = SVM linear—support vector classifier with linear kernels: the input data is non-linearly mapped to a higher dimensionality space, where a linear decision surface can be established [27];
3. SVC = SVM—support vector classifier with non-linear RBF kernels: the real problems tend not to have a linear solution, and SVM can handle this limitation by using nonlinear kernel functions such as Gaussian radial basis (RBF) [28];
4. LogisticRegression = LR—Logistic regression [29] is a linear model which can estimate the probability of a binary response using different factors;
5. LinearDiscriminantAnalysis = LDA—linear discriminant analysis [30]: a statistical supervised method that is based on the projection of data to a lower dimension to maximize the scatter between classes versus the scatter within each class. This projection facilitates the separation of the data;
6. DecisionTreeClassifier = DT—Decision Tree uses a series of decision rules inferred from the features as a tree of rules. Thus, the paths from root to leaf represent classification rules [31];
7. RandomForestClassifier = RF—Random forest [32] is an ensemble method that aggregates several decision trees (parallel trees). Each tree is generated using a bootstrap sample drawn randomly from the original dataset using a classification or regression tree (CART) method and the Decrease Gini Impurity (DGI) as the splitting criterion [33]. RF is characterized by low bias and low correlation between individual trees, and high variance;
8. XGBClassifier = XGB—XGBoost a tree-based ensemble method wherein weak classifiers are added to correct errors (sequential trees [34]). This classifier demonstrate excellent performances through the Kaggle competition projects [35].

### 2.5. ML Workflow

The features were standardized by removing the mean and scaling to unit variance, using the standard scaler in *scikit-learn*. A stratified 10-fold cross-validation was performed, preserving the percentages of samples for each class. As the dataset samples were not balanced, class weights were computed for each class using N/(k∗n_i_), where *N* is the total number of samples, *k* the number of classes and *n_i_* the number of samples belonging to the class *i.* This results in weights of 0.63778 for class 1 and 2.31448 for class 2. The model’s performance was measured using Area Under the Receiver Operating Characteristics (AUC).

Given the results obtained in the baseline, the workflow has continued only with the best model. From this point on, a feature selection was done using the mean impurity decrease, which is already implemented in sklearn. This metric is calculated using the weighted gini impurity decreases for all nodes, averaged over all trees [33]. Thus, a feature selection was done using ExtraTreesClassifier [36] with n_estimators = 100, class weights and 10-fold CV (see Feature-Selection.ipynb [37]). We chose this tree-based method to select the most important features because extra trees (sometimes named extreme random trees) offer a higher performance in the presence of noisy features [38]. Our custom feature selection algorithm keeps at least one feature for each experimental condition for drugs/compounds and nanoparticles, and the probability feature (if the automatic selector eliminates them).

The simplest PTML linear models will be the first classifiers to test for complex datasets with multiple BD characteristics [39,40]. We can approximate function values *f*(*v_ij_*(*d*) and *v_ij_*(*np*))*_calc_* for the i-th drug–nanoparticle pair in the j-th preclinical assay with multiple conditions of assay *c_j_*. As input, we used PT operators that can also be Box–Jenkins Moving Average (MA) operators [41,42]. PTML linear models have the following generic form:

(2) $$f{\left(v_{ij}\left(d\right),v_{ij}\left(np\right)\right)}_{calc}=\;a_{0}+a_{1}\cdot f{\left(v_{ij}\left(d\right),v_{ij}\left(np\right)\right)}_{expt}+\sum_{k=1,j=0}^{k_{max},j_{max}}\;a_{kj}\cdot \Delta D_{k}\left(d_{j},c_{j}\right)+\sum_{k=1,j=0}^{k_{max},j_{max}}\;b_{kj}\cdot \Delta D_{k}(np_{j},c_{j})$$

Additional results have been provided in order to explain the predictions with the best model using Shapley values [43] (SHAP_test.ipynb). All the scripts for baseline AUCs, feature selection and the final model are available as an open repository at https://github.com/d-bcarrue/NanoDrugsMalaria [37].

## 3. Results and Discussion

In the present work, we created a PTML model to predict the activity of organic compounds assembled of some nanoparticles used against malaria disease. In doing so, we expanded the idea behind Hansch’s analysis and searched models with applications to nanomedicine. As a proof-of-concept test, we investigated a huge number dataset of drugs downloaded from ChEMBL, and another dataset of nanoparticles. Those datasets contain (see materials and methods) the outcomes of many experimental pharmacological assays. 

The model supposes that the changes in drug–nanoparticle binding occur thanks to perturbations in the input boundary conditions of both nanoparticles and drugs. We focused only on a nanoparticle–drug/compound binding pseudo-constant (*v_ij_*_(*d*)_,*v_ij_*_(*np*)_), defined by us, to quantify the probability of a nanoparticle–drug/compound pair highly expressed against malarial activity. This PTML model begins with a reference value, $f\left(v_{ij_{d}},v_{ij_{np}}{)}_{expt}\right)$, and then adds the effects of perturbations in the structure of the compound or conditions of the assay, and the properties of the nanoparticle and its coating. Other input terms used here are the perturbation terms ΔLogP and ΔPSA, which are similar to the Moving Average (MA) functions utilized in the Box–Jenkins models in time series (Box and Jenkins, 1970). Example of MAs are the deviations of PSA and logP of compounds/drugs and nanoparticles from the expected values of these parameters for assays under the same conditions c_j_. For example, DLogP = LogP(m_i_) − LogP(c_j_), where LogP(c_j_) is the average of LogP(m_i_) for all molecules, m_i_, in the same assay with a set of conditions c_j_.

**Figure 2:**
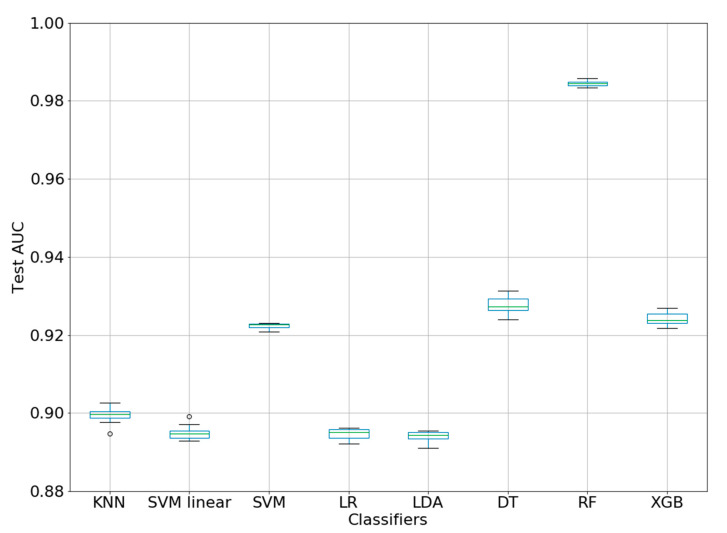
Box-plot for AUC values of ML classifiers (10-fold CV).

Using eight ML classifiers, the AUC values have been calculated (10-fold CV). The results are presented in Table 5. The best model was obtained with RF, and the AUC is 0.9844 ± 0.0007. Figure 2 represents the box-plot for the baseline AUC values of the ML methods (10-fold CV). The AUC values for the 10 splits have short ranges, especially RF. This suggests that the AUCs for all ML methods are stable within each fold. In addition, the high difference between the RF and the other methods (box-plots are far from overlapping) demonstrated that it is statistically significant.

In the next step, we reduced the number of features in order to improve the AUC of the RF model. Thus, a feature selection was done using ExtraTreesClassifier. The 27 features were selected from an initial 107:

- One np–compound pair feature: prob;
- 5 np features using 5 experimental conditions (c0-c4): np_DVnpu(c0), np_DUccoat(c1), np_DVnpu(c2), np_DPnpu(c3), np_DPnpu(c4);
- 21 drug/compound features using 7 experimental conditions (c0-c6): d_DMw(c0), d_DALOGP(c0), d_DPSA(c0), d_DMw(c1), d_DALOGP(c1), d_DPSA(c1), d_DMw(c2), d_DALOGP(c2), d_DPSA(c2), d_DMw(c3), d_DALOGP(c3), d_DPSA(c3), d_DMw(c4), d_DALOGP(c4), d_DPSA(c4), d_DMw(c5), d_DALOGP(c5), d_DPSA(c5), d_DMw(c6), d_DALOGP(c6) and d_DPSA(c6).

Remarkably, this is the first model combining both Perturbation Theory and MAs in a QSBR study of relevant nanoparticle–drug/compound pairs used as an antimalarial delivery system. We determined the more relevant perturbations under different experimental conditions, c_j_, related to the antimalarial property by using a RF. Casually, in this model most of the used operators are of PSA and ALOGP type. Therefore, they measure only perturbations in the value of ALOGP with respect to other subsets, c_j_, of drugs and nanoparticles. ALOGP is a relevant parameter used in medicinal chemistry because it is related to lipophilicity and the capacity to cross biological membranes.

**Figure 3:**
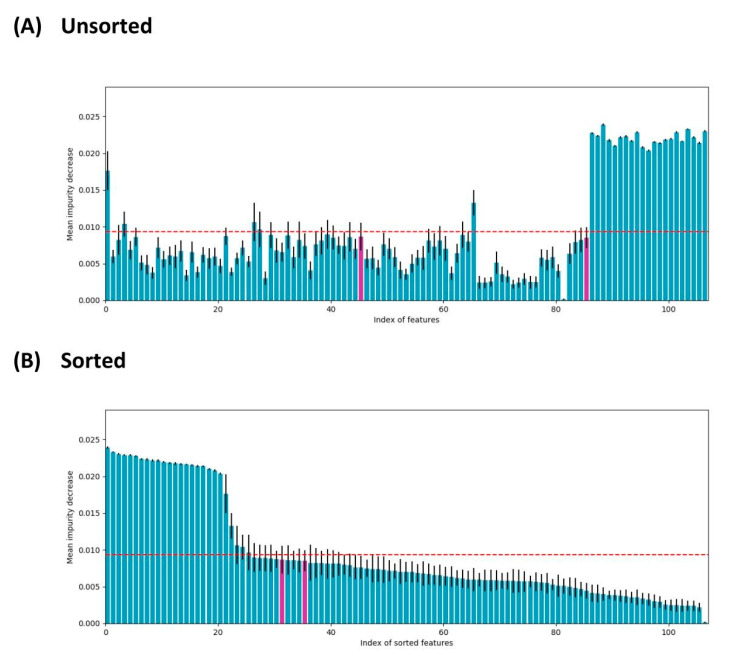
Feature selection using ExtraTrees: mean impurity decrease by feature, in original order and in descending order; pink bars represent features reintroduced after initial filtering.

Figure 3 shows the mean impurity reduction for each of the features in both the original order and the sorted. This mean impurity decrease was obtained using an Extra Trees classifier with 100 trees and weighted classes, and this model was applied in a stratified 10-fold CV. The horizontal dashed line indicates the threshold used as a selection filter. After checking that the probability feature is present, since this is strictly necessary in Perturbation Theory, we check that all experimental conditions are reflected in the selected subset. After filtering, the experimental conditions, c2 and c4, of the nanoparticles were not included. Therefore, we selected the characteristic with the highest mean impurity decrease for both experimental conditions, and added it to the previous selection, marking them in pink. The unsorted plot presents the features on the x-axis in the order they were presented into the dataset. For a better comparison of the selected feature mean impunities (the ones above the cutoff), the ordered version of the plot was presented too.

Therefore, with only 27 selected features (from an initial 107), the mean test AUC for the RF classifier increased to 0.9921 ± 0.000244 (from 0.9844 ± 0.0007). This model shows a very good performance for a PTML model.

The feature selection showed that the current classifier prefers perturbations (MAs) of the logarithmic term of the octanol/water partition coefficient (ALOGP), polar surface area (PSA) and molecular weight (Mw) for compounds/drugs under all experimental conditions, such as activity type (c0), organism (c1), cell name (c2), assay organism (c3), assay strain (c4), type of curation (c5) and assay type (c6). In the case of the nanoparticles, the model selected the perturbations of the average of atomic Van der Waal volume for all atoms in the np (Vnpu) with activity type (c0) and with shape (c2), unsaturation count (Uccoat) in cell line (c1), atomic polarizability (Pnpu) with assay medium (c3) and surface coating (c4). Thus, we can conclude that the perturbations of the following molecular descriptors under different experimental conditions are important for anti-malaria drug-decorated nanoparticles: polarity of both components/drugs and nanoparticles, mass of compounds/drugs, volume, shape and coating unsaturation of nanoparticles. 

A linear model is easily interpreted, but it is not always the most accurate model. Therefore, the complex models use different tools in order to avoid a “black box” model. Shapley values and SHAP (Shapley Additive explanations) values are the proposed solution for the best RF model. The Shapley values represent the average of the marginal contribution across all permutations, a method for quantifying the contribution of the features to the final model. Thus, the SHAP method is able to explain the output of a machine learning model by:

- global interpretability: how much each feature contributes, either positively or negatively, to the output variable;
- local interpretability: each case/instance gets its own SHAP values in order to explain why a case has a specific prediction, and the contribution of the features to this instance.

**Figure 4:**
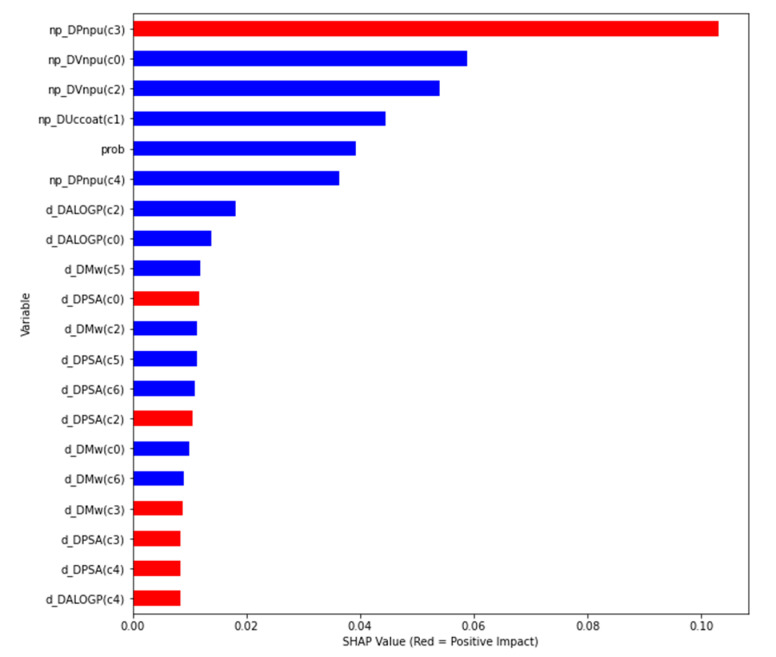
Feature impact on the output variable for the best model based on mean SHAP values.

The global interpretability is presented by the correlation of the features with the output variable or the positive/negative impact using SHAP values (Figure 4). The ordered average impact of the features on the model output for each class, and the local interpretability for different instances/cases, are included in the GitHub repository with the new script (SHAP_test.ipynb).

This figure is presenting:

- Feature importance using SHAP values: the variables are ranked in descending order;
- Impact on the prediction value using SHAP values on x-axis;
- Color shows whether that variable has a positive (in red) or a negative (in blue) impact on the output variable.

Thus, we can observe that the nanoparticle perturbation of molecular descriptors under experimental conditions has a high impact on the model prediction for anti-malaria drug/compounds carriers. These include perturbation of atomic polarizability (Pnpu), the average of atomic Van der Waal volume for all atoms (Vnpu) and unsaturation of coating (Uccoat). For the compounds/drugs, ALOGP has more impact than mass weight and PSA. Thus, we confirm that the molecular properties linked to polarity have the highest impact on the anti-malaria drug/compound–nanoparticle carriers. Atomic polarizability of nanoparticles has a more positive impact on the model output, and the volume of nanoparticles has only a negative impact: the optimal anti-malaria drug–np carriers should consider nanoparticles with high atomic polarizability but small volume. In addition, the compounds/drugs should have higher polar surface areas (PSA) with a positive impact, and smaller weight mass with a negative impact, on the model output.

## 4. Conclusions

By combining Perturbation Theory ideas with Hansch’s QSAR analysis and information fusion, we developed a multi-target PTMLIF model that is useful in classifying drugs based on their constant binding to many different nanoparticles and their capacity to act against plasmodium, which is the cause of malaria in humans. This model can help us to save experimental resources and time, since it allows the determination of which drug-decorated nanoparticles would be useful and which would not. In this way, we can prove only those with the highest probability of being active. The transformed features of drugs and nanoparticles have been used as input for eight Machine Learning methods. The best classification model has been obtained using Random Forest with only 27 selected features of drugs and nanoparticles in all the experimental conditions considered. The mean test AUC was 0.9921 ± 0.000244 (10-fold CV). The performance of the RF model demonstrated the power of the information fusion of the experimental-based features of drugs and nanoparticles for the prediction of probability, related to nanoparticle–drug/compound antimalarial activity. All the calculations can be reproduced using the scripts and dataset included in an open GitHub repository at https://github.com/d-bcarrue/NanoDrugsMalaria.

## Figures and Tables

**Table 1:** CHEMBL assay conditions (selected examples).

c_0_ = Parameter	n_j_^a^	c_0_ = Parameter	n_j_
IC_50_ nM	30,981	IC_50_ ug·mL^−1^	4914
EC_50_ nM	10,337	CC_50_	11,629
**c_1_ = Organism**	**n_j_**	**c_1_ = Organism**	**n_j_**
Plasmodium falciparum D6	564	Plasmodium falciparum K1	6066
Plasmodium falciparum	35,463	Plasmodium yoelii yoelii	36
Plasmodium berghei	471	Plasmodium cynomolgi	15
**c_2_ = Cell name**	**n_j_**	**c_2_ = Cell name**	**n_j_**
Erythrocyte	677	Huh-7	118
FM3A	14	L6	85
HeLa	19	MRC5	23
Hepatocyte	28	Oocyte	5
HepG2	123	Vero	156
**c_3_ = Assay organism**	**n_j_**	**c_3_ = Assay organism**	**n_j_**
Plasmodium falciparum	31,587	Plasmodium berghei ANKA	6
Plasmodium falciparum D10	1147	Plasmodium falciparum 3D7	2606
Plasmodium falciparum FcB1/Columbia	330	Plasmodium falciparum NF54	938
Plasmodium berghei	461	Plasmodium falciparum FCR-3/Gambia	31
**c_4_ = Assay Strain**	**n_j_**	**c_4_ = Assay Strain**	**n_j_**
W2mef	39	W2	8591
NF54	929	TM91C235	474
W2/Indochina	31	VS1	25
W2-Mef	16	TM90C2B	68
**c_5_ = Curated by**	**n_j_**	**c_5_ = Curated by**	**n_j_**
Autocuration	38,150	Expert	2794
Intermediate	5288		
**c_6_ = Assay Type**	**n_j_**	**c_6_ = Assay Type**	**n_j_**
F	46,179	B	53

^a^ n_j_ indicates the number of samples for each of the conditions.

**Table 2:** Compound activity parameters (c_0_).

c_0_ = Activity (Units)	Statistical Parameters ^a^						
	<LogP>	<PSA>	n_0_	n_1_	p_1_	d	Cutoff
IC_50_ nM	4.128024	72.6311	30,981	8954	0.289	−1	100.0
EC_50_ nM	4.2390887	67.1602	10,337	1437	0.139	−1	100.0
IC_50_ ug.mL^−1^	4.0724379	75.0632	4914	4889	0.994	−1	325.0
CC_50_ nM	4.0650589	67.7534	11,629	11,608	0.998	1	100.0

^a^ Parameters <LogP> and <PSA> = Average value of LogP and PSA for all drugs m_i_ with value reported in ChEMBL dataset. These parameters are needed for the moving average calculation. Other parameters: n_0_ = number of compounds that shown each different activity, n_1_ = number of compounds considered as positive, p_1_ = n_1_/n_0_ probability of a compound being considered positive, d = −1, 0, 1 is the desirability of the parameter, cutoff = limit for the compound being treated as active or not.

**Table 3:** Decorated Nanoparticles assay conditions (selected examples).

c_0_ = Parameter	n_j_^a^	c_0_ = Parameter	n_j_
IC_50_ nM	29	CC_50_	113
EC_50_ nM	30		
**c_1_ = Cell line**	**n_j_**	**c_1_ = Cell line**	**n_j_**
A549 (H)	23	BRL 3A (R)	4
Lycopersicon esculentum	16	3T3 (M)	9
HepG2 (H)	15	CaCo-2 (H)	6
**c_2_ = Shape**	**n_j_**	**c_2_ = Shape**	**n_j_**
Spherical	61	Elliptical	21
Irregular	3	Pseudo-spherical	8
Slice-shaped	3	Polyhedral	3
Needle	2	Pyramidal	10
Rod	9		
**c_3_ = Assay Medium**	**n_j_**	**c_3_ = Assay Medium**	**n_j_**
Dry	118	RPMI	3
H_2_O	44	1% Triton X-100/H_2_O	3
DMEM	3	H_2_O/TMAOH	1
**c_4_ = Surface coating**	**n_j_**	**c_4_ = Surface coating**	**n_j_**
UC	125	11-mercaptoundecanoic acid	3
PEG-Si(OMe)_3_	8	PVP	4
PVA	1	Propylamonium fragment	4
Sodium citrate	17	Undecylazide fragment	2

^a^ n_j_ = the number of samples for each of the conditions; IC_50_ = the half maximal inhibitory concentration; EC_50_ = the concentration of a drug that gives half-maximal response; CC_50_ = the ratio of the 50% cytotoxic concentration; A549 (H) = Lung carcinoma cells; HepG2 (H) = human liver cancer cells; BRL 3A (R) = Buffalo Rat Liver cells; 3T3 (M) = Fibroblast cells; CaCo-2 (H) = human colon carcinoma cells; DMEM = Dulbecco’s modified eagle medium; RPMI = Roswell Park Memorial Institute medium; TMAOH = Tetramethylammonium hydroxide; UC = uncoated; PEG-Si(OMe)3 = trimethoxysilyl poly(ethylene glycol); PVP = Polyvinylpyrrolidone; PVA = Polyvinyl alcohol.

**Table 4:** Nanoparticle activity parameters (c_0_).

c_0_ = Activity (Units)	Statistical Parameters ^a^						
	<LogP>	<PSA>	n_0_	n_1_	p_1_	d	Cutoff
EC_50_ uM	1.66	18.02	30	27	0.9	−1	25,422
IC_50_ uM	3.24	38.79	29	21	0.7241	−1	18,714
CC_50_ uM	1.63	24.97	113	21	0.1858	1	3099

^a^ Parameters <LogP> and <PSA> = Average value of LogP and PSA for all nanoparticles m_i_. These parameters are needed for the moving average calculation. Other parameters: n_0_ = number of decorated nanoparticles that shown each different activity, n_1_ = number of nanoparticles considered as positive, p_1_ = n_1_/n_0_ probability of a nanoparticle being considered positive, d = −1, 0, 1 is the desirability of the parameter, cutoff = limit for the DNPs being treated as active or not.

**Table 5:** Area Under the Receiver Operating Characteristics (AUC) for baseline classification models.

ML Method.	Classifier	AUC Mean + sd
KNN	KNeighborsClassifier	0.8994 ± 0.0022
SVM linear	SVC(linear)	0.8949 ± 0.0019
SVM	SVC(rbf)	0.9223 ± 0.0007
LR	LogisticRegression	0.8946 ± 0.0013
LDA	LinearDiscriminantAnalysis	0.8939 ± 0.0015
DT	DecisionTreeClassifier	0.9277 ± 0.0021
RF	RandomForestClassifier	0.9844 ± 0.0007
XGB	XGBClassifier	0.9242 ± 0.0017

KNN = k-nearest neighbors; SVM linear = support vector classifier with linear kernels; SVM = support vector classifier with non-linear kernels; LR = Logistic regression; LDA = linear discriminant analysis; DT = Decision Tree; RF = Random forest; XGB = XGBoost.
